# Analysis of GCRV Pathogenesis and Therapeutic Measures Through Proteomic and Metabolomic Investigations in GCRV-Infected Tissues of Grass Carp (*Ctenopharyngodon idella*)

**DOI:** 10.3390/ijms252111852

**Published:** 2024-11-04

**Authors:** Juhong Xie, Zhihui Jia, Yangyang Li, Lanjie Liao, Zuoyan Zhu, Yaping Wang, Rong Huang

**Affiliations:** 1 State Key Laboratory of Freshwater Ecology and Biotechnology, Institute of Hydrobiology, Chinese Academy of Sciences, Wuhan 430072, China; xiejuhong@ihb.ac.cn (J.X.);; 2 College of Advanced Agricultural Sciences, University of Chinese Academy of Sciences, Beijing 100049, China; 3 College of Fisheries and Life Science, Dalian Ocean University, Dalian 116023, China

**Keywords:** *Ctenopharyngodon idella*, grass carp reovirus, DIA proteomics, untargeted metabolomics, fumaric acid

## Abstract

Hemorrhagic disease caused by grass carp reovirus (GCRV) infection is a major problem affecting the grass carp aquaculture industry. Therefore, inhibiting the spread of GCRV infection is of great economic significance. Herein, we sequenced five tissues (gill, liver, intestine, kidney, and muscle) from grass carp before and after GCRV infection using data-independent acquisition proteomic and untargeted metabolomic technologies, and quantitatively identified 10,808 proteins and 4040 metabolites. Then, we analyzed the differentially expressed proteins (DEPs) and metabolites (DEMs) before and after GCRV infection in the five tissues. Gene ontology analysis revealed that the five tissue DEPs were enriched in metabolic, including carbohydrate and lipid metabolic processes. Chemical taxonomy analysis showed that the categories of DEMs mainly included carbohydrates and lipids, such as fatty acids, glycerophospholipids, steroids, and their derivatives. Both the proteomic and the metabolomic data showed that GCRV affected the carbohydrate and lipid metabolism in the host. Shared pathway analysis was performed at both the protein and metabolic levels, showing significant enrichment of the glycolysis and pentose phosphate pathways (*p* < 0.001). Further analysis of glycolysis and pentose phosphate pathway inhibitors revealed that these two pathways are important for GCRV replication. As the kidney was the most affected among the five tissues, we analyzed the butanoate metabolism in the kidney, which revealed that most of the differentially expressed proteins and differently expressed metabolites in the butanoate metabolism were related to the TCA cycle. Further investigation showed that fumaric acid, an intermediate product in the TCA cycle, significantly inhibited GCRV replication in the CIK cells (*p* < 0.001), and that this inhibitory effect may be related to its induction of interferon system activation. The addition of fumaric acid to feed increased the survival rate of juvenile grass carp by 19.60% during GCRV infection, and protected the tissues of those infected with GCRV, making it a potential anti-GCRV feed additive. Our results provide new perspectives on GCRV pathogenesis and antiviral strategies for grass carp.

## 1. Introduction

Grass carp (*Ctenopharyngodon idella*) is the fish species with the highest aquaculture production in China, being farmed throughout the country. The total aquaculture production of grass carp in China in 2022 was 5,904,800 tons, yielding in significant economic benefits. However, grass carp hemorrhagic disease (GCHD), caused by grass carp reovirus (GCRV), represents a major bottleneck in the grass carp aquaculture industry. According to the “Report on the Health Status of China’s Aquatic Animals in 2023”, the economic loss of grass carp aquaculture due to this disease was CNY 1.9 billion in 2022, of which GCHD was the main disease monitored in grass carp aquaculture. GCRV can infect tissues such as the gills, liver, intestine, kidney, and muscles of grass carp, resulting in clinical conditions such as multi-organ hemorrhages throughout the abdomen, as well as muscle hemorrhages [1]. Studying the response mechanisms of various grass carp tissues to GCRV infection will be important in understanding the pathogenic mechanisms of GCRV.

Omics techniques have been used to study the biological responses of grass carp to GCRV infection from a holistic perspective. Indeed, transcriptomic studies showed that grass carp activated downstream immune responses via three innate immune signaling pathways: Toll-like receptors (TLRs), RIG-I-like receptors (RLRs), and nucleotide-binding and oligomerization domain-like receptors (NODs) following infection with GCRV. Nucleotide-binding and oligomerization domain-like receptors (NOD), which function in three innate immune signaling pathways, activate downstream immune responses [2]. The kidney is an important immune organ in grass carp, while differentially expressed genes (DEGs) in the kidney of grass carp before and after GCRV infection were found to be predominantly enriched in the immune response pathway; all interferon genes related to the immune response were significantly changed, while the complement and coagulation cascade pathways were also significantly upregulated [3]. Subsequently, the DEGs and their associated signaling pathways in the pre-, mid-, and late stages of GCRV infection were elaborated using grass carp kidney cells (*Ctenopharyngodon idella* kidney, CIK) as a model [4]. Recently, transcriptome studies in the muscle of resistant and susceptible grass carp identified 40 differential immune-related genes and 28 differential interferonstimulating genes (ISGs) related to the interferon (IFN) pathway [5]. In further studies using proteomics, viral invasion was found to affect the biosynthesis and cellular metabolic processes of CIKs, including the ribosomal pathway, amino acid biosynthesis, fructose and mannose metabolism, pentose phosphate pathway, galactose metabolism, glycolysis, and carbon metabolism [6]. Similar to the results of transcriptomic studies, GCRV was also found to activate the RIG-I receptor and its downstream pathways in the spleen of grass carp at the protein level. Additionally, GCRV can activate Janus kinase-signal transducer and activator of transcription (JAK-STAT) pathway and its related lipid metabolic pathways and necrotic apoptosis, causing severe inflammatory responses and metabolic disorders [7]. The rare minnow (*Gobiocypris rarusis*) is an ideal model for studying GCRV infections. Isobaric tags for relative and absolute quantitation (iTRAQ)-based proteomic studies have shown that this virus induces the upregulation of ATP synthesis and ubiquitination-proteasomal processes, as well as the downregulation of the carbon metabolism in the spleen of the rare minnow [8]. In recent years, researchers have begun to investigate the mechanisms underlying the age-dependent susceptibility of grass carp to GCRV infection at the metabolic level, finding that tryptophan, purine, and the glycerophospholipid metabolism are the main metabolic processes that differ between susceptible and resistant fish [9]. Differential metabolites and metabolic pathways in the spleen and hepatopancreas of rare minnows in response to GCRV infection with different toxicities have also been analyzed [10]. Previous studies predominantly focused on the CIKs, spleen, and kidney of grass carp or rare minnows. However, GCRV can invade multiple organs in grass carp, meaning that the use of new sequencing technology to conduct multi-omics analysis of multiple organs in grass carp could provide a more accurate and comprehensive understanding of the mechanism of the organismal response of grass carp to GCRV infection.

Data-independent acquisition (DIA) proteomics is a recently-developed holistic proteomics strategy based on mass spectrometry (MS) characterized by broad protein coverage, and high reproducibility and accuracy. Combining DIA proteomics with other technological advances, such as sample preparation and computational data analysis can further improve the analytical performance [11]. Untargeted metabolomics is an unbiased metabolomics analysis technique used to discover new biomarkers or differential metabolites, and its ability to detect a large number of metabolite signals simultaneously is important for understanding biological functions and diseases [12]. The integrative analysis of both proteomic and metabolomic data to identify differential proteins and metabolites that share a certain type of metabolic pathway or have the same trend for change can further help to reveal the mechanism underlying the molecular regulation of phenotypic changes, and make the analysis more comprehensive [13,14]. For example, a combined proteomic and metabolomic analysis of human ischemic and dilated cardiomyopathy allowed the identification of the shared metabolic pathways of these two cardiomyopathies, as well as the metabolites related to disease and sex [13]. Further, by using metabolomic, proteomic, and genomic technologies to analyze the tumor tissues of mice on high-fat diets, researchers have shown that obesity impairs the tumor microenvironment of mice with respect to the number of CD8+ T-cells and anti-tumor activity, which in turn accelerates tumor growth [14].

In this study, we performed DIA proteomics and untargeted metabolomic analyses of five tissues (gill, liver, intestine, kidney, and muscle) of grass carp before and after GCRV infection. In summary, our results showed that glycolysis and the pentose phosphate pathway of carbohydrate metabolism were critical for GCRV replication. We further investigated the butanoate metabolism in the kidney, the main tissue responding to GCRV infection, and found that fumaric acid exerts antiviral effects; thus, we explored the feasibility of fumaric acid as a feed additive.

## 2. Results

### 2.1. GCRV Is Able to Infect Five Tissues of Grass Carp

Following GCRV infection, the viral load in the five grass carp tissues was detected at the time of peak mortality (Supplementary Figure S1). The results showed that GCRV was able to infect all five tissues, with the highest viral load in the muscle, followed by the intestine, kidney, gill, and liver.

### 2.2. DIA Proteomics

**Figure 1:**
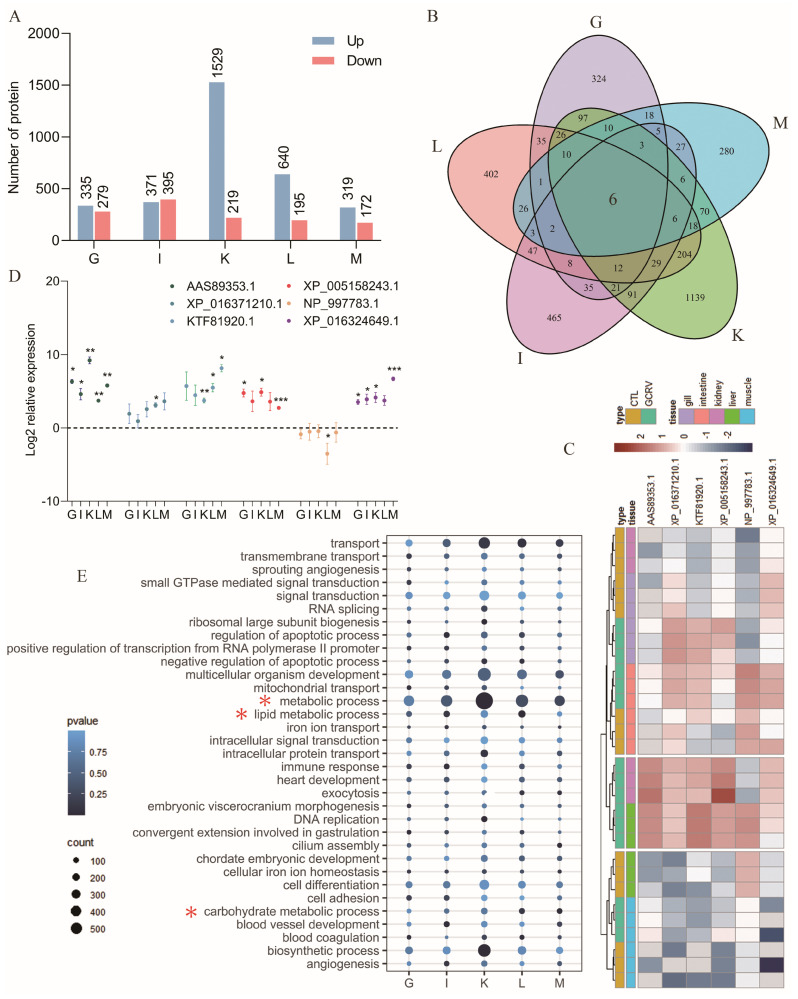
Proteomic analyses of five tissues. (**A**) Number of up- and down-regulated DEPs in the five tissues. (**B**) Venn diagram of tissue-shared DEPs. (**C**) Expression patterns of tissue-shared DEPs in each tissue. (**D**) qPCR validation of the expression pattern of tissue-shared DEPs in each tissue, names above the dashed line represents up-regulation and below the dashed line represents down-regulation. (**E**) Shared GO biological processes enriched in the five tissues DEPs. (* *p* < 0.05, ** *p* < 0.01, *** *p* < 0.001).

Mass spectrometry data were collected from 30 samples in DIA mode, allowing the quantification of 114,015 peptides and 10,808 proteins. The information for all the proteins identified before and after the GCRV infection of the five tissues was shown in the Supplementary Tables S1–S5. The samples were initially assessed for inter- and intragroup differences using principal component analysis (PCA) (Supplementary Figure S2), which revealed a large difference in the kidneys before and after viral infection. Subsequently, the DEPs in each tissue were analyzed, and the results showed that the kidneys had the highest number of DEPs, consistent with the results of PCA. A greater number of upregulated proteins were found in the gills, liver, kidney, and muscle, whereas more downregulated proteins were found in the intestine (Figure 1A). 

To explore the key proteins and biological functions affected by GCRV infection, we analyzed the shared DEPs and biological processes in five tissues. Overall, we identified six shared DEPs among the five tissues following viral infection (Figure 1B). Figure 1C shows the accession numbers of six proteins in the non-redundant protein database. Except for NP_997783.1 (CTRB1, FC = 0.46, *p* < 0.05) and XP_016324649.1 (OLFM4, FC = 0.32, *p* < 0.001), which were down-regulated in the gills and up-regulated in other tissues, the other proteins were up-regulated in all five tissues following infection. For the preliminary validation of the proteomic data, we performed qPCR for the six shared DEP genes at the transcriptional level. The results showed that CTRB1 was significantly downregulated in the liver (*p* < 0.05), with no differences in other tissues, which was inconsistent with the proteomic data. OLFM4 was significantly up-regulated in the gills (*p* < 0.05), whereas the proteomic data showed its downregulation. These phenomena may be explained by the existence of a series of post-transcriptional modifications or regulations of these two proteins, making their expression patterns slightly different at the transcriptional and protein levels. The expression patterns of the other four genes in the five tissues were upregulated, which agreed with the proteomic data (Figure 1D). The biological processes of the DEPs in the five tissues were enriched in metabolic and explicit secondary metabolic processes, including lipid and carbohydrate metabolism (Figure 1E).

### 2.3. Untargeted Metabolomics

**Figure 2:**
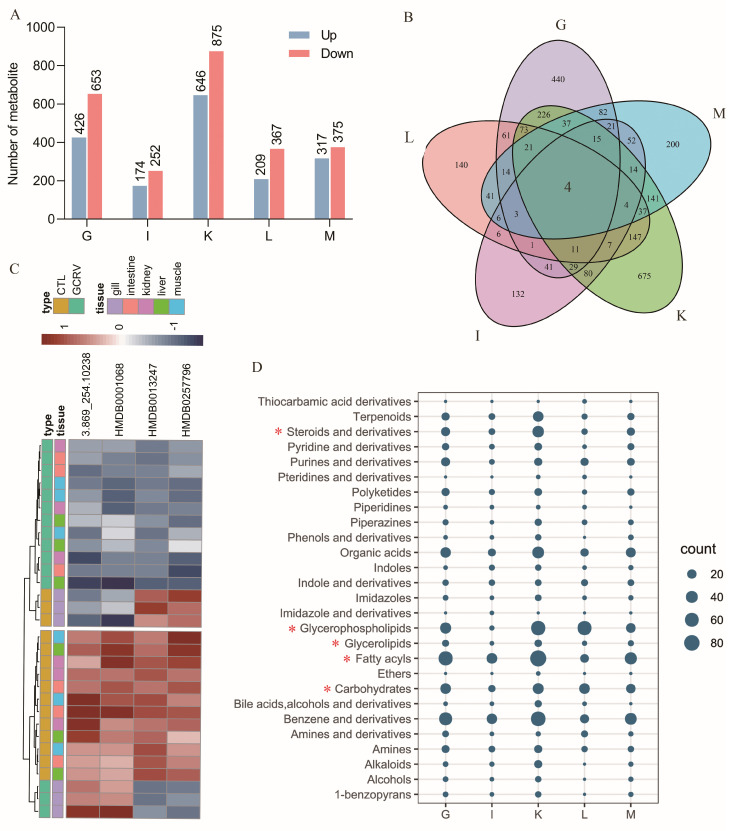
Metabolomic analyses of the five extracted tissues. (**A**) Number of up- and down-regulated DEMs in the five tissues. (**B**) Venn plot of tissue-shared DEMs. (**C**) Tissue-shared DEMs expression patterns in each tissue. (**D**) Shared classes of tissue differential metabolites in the five tissues DEMs.

Untargeted metabolomic sequencing was performed using an UPLC-MS, and 4040 metabolites were identified. The information of all the metabolites identified before and after the GCRV infection of the five tissues is shown in Supplementary Tables S6–S10. Intergroup differences were assessed using PCA, and the tissues before and after infection were divided into two groups, indicating a good infection effect (Supplementary Figure S2). The DEMs in each tissue were subsequently analyzed, and, consistent with the proteomic data, the results showed that the kidneys had the highest number of DEMs. The primary difference was that there were more downregulated than upregulated metabolites in each tissue, with the kidney having the highest number of up and downregulated metabolites at 646 and 875, respectively, and the intestine having the lowest number of metabolites at 174 and 252, respectively (Figure 2A).

To further explore the key metabolites and metabolite classes affected by GCRV infection, we analyzed the shared DEMs and their classes in the five tissues. Overall, we identified four shared DEMs in the five tissues following viral infection (Figure 2B); the accession numbers of these four proteins in the BMDB database, the mzCloud database, and the ChemSpider online database are listed in Figure 2C, respectively. With the exception of 3.869_254.10238 (methyl dimethoxybenzoate, FC = 3.40, *p* < 0.01) and HMDB0001068 (heptose 7-phosphate S7P, FC = 1.35, *p* < 0.05), which were up-regulated in the gills, the expression patterns of the other metabolites were essentially the same, with down-regulation occurring after infection (Figure 2C). The shared categories of differential metabolites in the five tissues after infection were mainly related to lipid and carbohydrate metabolism, including fatty acids, glycerophospholipids, carbohydrates, glycerolipids, and steroids and their derivatives (Figure 2D).

### 2.4. KEGG Pathway Analysis

**Figure 3:**
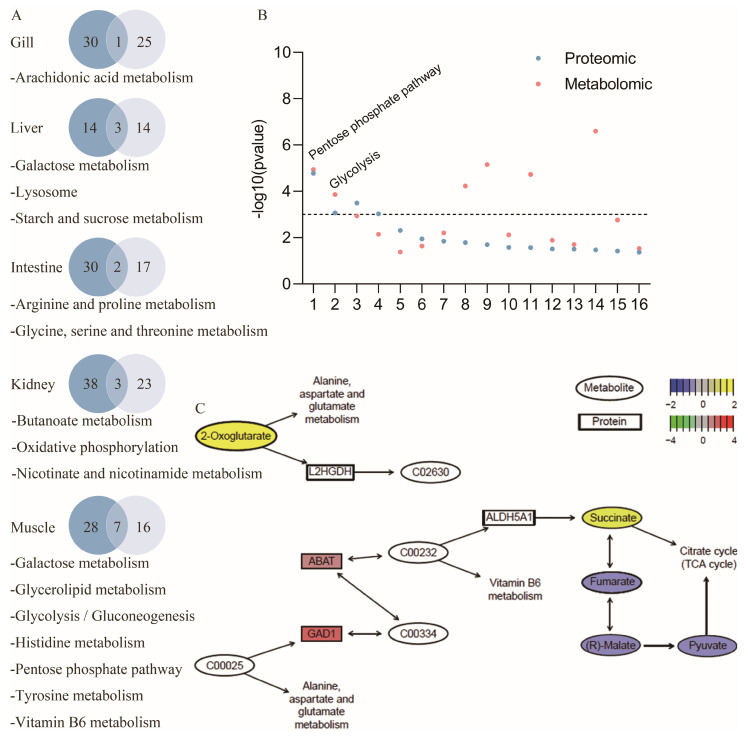
Analysis of proteomic and metabolomic KEGG pathways in the five tissues. (**A**) Number of pathways and pathway names with significant changes in the proteome (blue) and metabolome (gray). (**B**) *p* values of pathways with significant changes at the protein and metabolic levels; symbols above the dashed line represent *p* < 0.001. (**C**) Changes in proteins and metabolites associated with the TCA cycle of butanoate metabolism in kidney.

KEGG enrichment analysis was performed on both DEPs and DEMs in each tissue at the protein and metabolite levels. Pathways with *p* < 0.05 were selected for analysis to identify shared pathways at the protein and metabolite levels in each tissue. Consequently, 16 pathways were identified, including those involved in carbohydrate metabolism (*n* = 6), immune-related amino acid metabolism (*n* = 4), lipid metabolism (*n* = 2), metabolism of cofactors and vitamins (*n* = 2), energy metabolism (*n* = 1), and transporter catabolism (*n* = 1). In addition, the specific metabolic pathways affected by GCRV differed among the different tissues: in the gill, GCRV affected one lipid metabolic pathway, arachidonic acid metabolism; in the intestine, GCRV affected the amino acid metabolism, including two pathways, arginine and proline metabolism, and glycine, serine, and threonine metabolism; in the kidney, GCRV affected three pathways, butanoate metabolism, nicotinic acid and nicotinamide metabolism, and oxidative phosphorylation belonging to the carbohydrate metabolism, cofactor and vitamin metabolism, and energy metabolism, respectively; in the liver, GCRV affected galactose metabolism, starch and sucrose metabolism, and the lysosomal pathway, which are categorized as the carbohydrate metabolism and transporter catabolism pathways, respectively; GCRV affected seven shared pathways in the muscle, including the galactose metabolism, glycerophospholipid metabolism, glycolysis, histidine metabolism, pentose phosphate pathway, tryptophan metabolism, and vitamin B6 metabolism, which belong to the carbohydrate metabolism, lipid metabolism, amino acid metabolism, and metabolism of cofactors and vitamin pathways, respectively (Figure 3A). The *p* values of the 16 metabolic pathways obtained were further analyzed at the protein and metabolic levels, and the enriched pathways of glycolysis and pentose phosphate metabolism were both found to be extremely significant (*p* < 0.001), with a higher confidence level in comparison (Figure 3B). These results indicate that although GCRV affects different metabolic pathways in various host tissues, it is predominantly involved in carbohydrate metabolism.

The kidneys were selected as the tissue for further analyses, as they had the highest number of DEPs and DEMs. Of the three metabolic pathways, only the butanoate metabolism belonged to the carbohydrate metabolism, while 14 DEPs and DEMs were enriched in the butanoate metabolism, of which seven were concentrated in the tricarboxylic acid cycle. For example, 4-aminobutyric acid aminotransferase (ABAT, FC = 2.43, *p* < 0.05) and glutamic acid decarboxylase 1 (GAD1), which promote the TCA cycle, were up-regulated (FC = 4.87, *p* < 0.0001). The TCA cycle intermediates also showed changes; 2-oxoglutarate (FC = 7.04, *p* < 0.001) and succinate (FC = 1.48, *p* < 0.05) were up-regulated, and fumarate (FC = 0.55, *p* < 0.0001), maleate (FC = 0.54, *p* < 0.01), and pyruvate (FC = 0.47, *p* < 0.05) were downregulated (Figure 3C).

### 2.5. Glycolysis and the Pentose Phosphate Pathways Are Important for GCRV Replication

**Figure 4:**
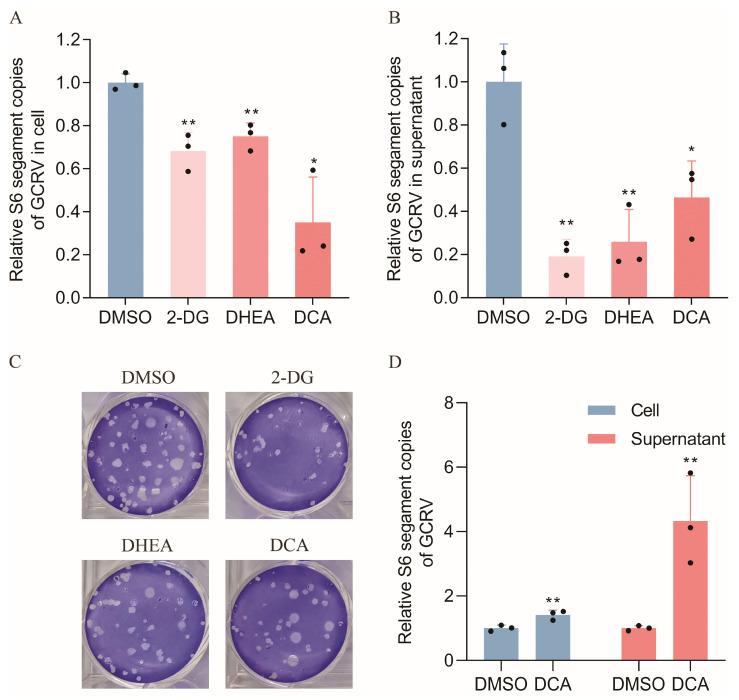
Glycolysis and the pentose phosphate pathway are important for GCRV replication. (**A**) Relative expression level of GCRV in CIK cells following 4 h of pretreatment with inhibitors of glycolysis (2-DG) and the pentose phosphate pathway (DHEA), and a TCA cycle enhancer (DCA). (**B**) Relative expression level of GCRV in the supernatant of CIK cells after 4 h pretreatment with 2-DG, DHEA, and DCA. (**C**) Number of empty spots produced by crystal violet staining of GCRV in CIK cells after 2-DG, DHEA, and DCA treatment. (**D**) DCA treatment was performed 6 h after GCRV infection, and the relative expression level of GCRV in intracellular and cell supernatants was identified. (* *p* < 0.05, ** *p* < 0.01).

As KEGG pathway enrichment of the glycolysis and pentose phosphate metabolism reached highly significant levels at both the protein and metabolic levels (*p* < 0.001), we treated CIK cells with the glycolysis inhibitor 2-DG and the pentose phosphate pathway inhibitor DHEA, and then infected them with GCRV to observe the effect on GCRV replication. The effects of different concentrations of inhibitors on CIK cell viability are presented in Supplementary Figure S3; the final concentrations of 2-DG and DHEA used were 0.8 mg/mL and 20 µM, respectively. The results showed that the GCRV content in both the intracellular and supernatant was significantly lower than that in the control group following treatment with the two inhibitors (Figure 4A,B). After 2-DG and DHEA treatment, we performed crystal violet staining to detect the number of vacuoles produced by GCRV in CIK cells. The results of this assay showed that the number of vacuoles in the inhibitor-treated group was fewer than that in the control group (Figure 4C), suggesting that the inhibition of glycolysis and pentose phosphate metabolism by 2-DG and DHEA was unfavorable to GCRV replication. These results suggest that host glycolysis and pentose phosphate metabolic pathways play important roles in GCRV replication.

Since the DEPs biological processes, DEM classes, and KEGG pathways of the five tissues were all co-enriched for lipid metabolism, while the TCA cycle is closely related to the synthesis of fatty acids required for viral replication, we also treated cells with the TCA cycle promoter DCA, to observe the effect of the lipid metabolism on GCRV replication. The effects of different concentrations of DCA on CIK cell ability are shown in Supplementary Figure S3, and the final concentration of DCA used was 20 µM. The results show that treatment of cells with DCA before GCRV infection (4 h before infection) resulted in a decrease in the GCRV content in cells and supernatants (Figure 4A,B), while the number of empty spots detected by crystal violet staining was lower than that of the control group (Figure 4C). However, the treatment of cells with DCA after GCRV infection (6 h after GCRV infection) was found to significantly promote GCRV replication in the cells and cell supernatants (Figure 4D). These results indicate that the intermediates of the TCA cycle are complex, and that promoting the TCA cycle and lipid metabolism at different times may have different effects on GCRV replication.

### 2.6. Antiviral Function of Fumaric Acid

**Figure 5:**
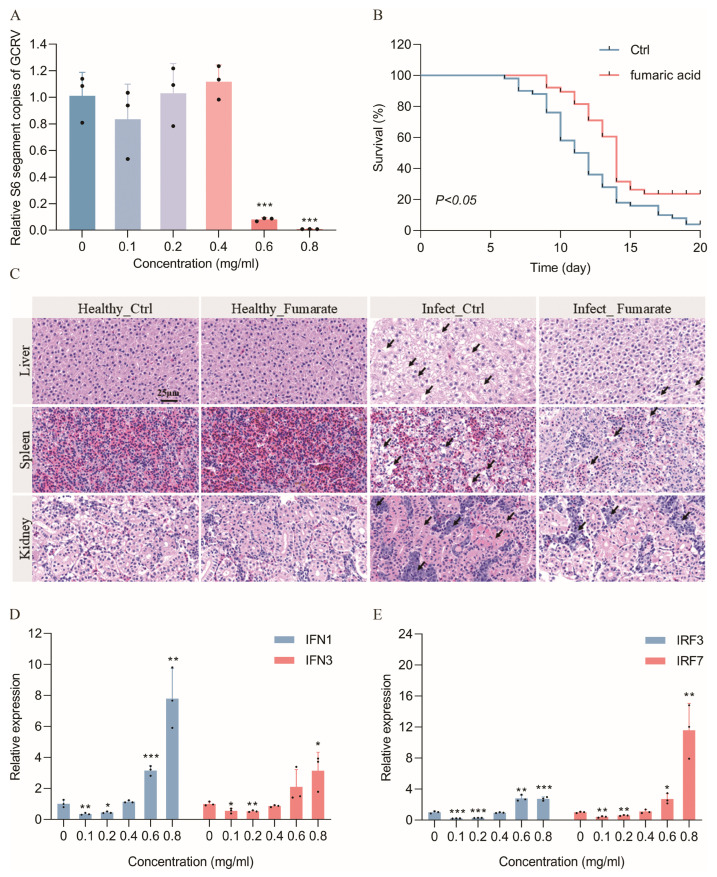
Antiviral effects of fumaric acid. (**A**) Effect of different concentrations of fumaric acid on GCRV replication in CIK cells. (**B**) Survival curves of GCRV-infected juvenile grass carp after feeding with a fumaric acid or normal diet for 14 days. (**C**) HE-stained tissue sections of the liver, spleen and kidney of juvenile grass carp fed fumaric acid diet and normal diet before and after GCRV infection. (**D**) Induction of IFN1 and IFN3 by different concentrations of fumaric acid, detected after GCRV infection of CIK cells. (**E**) Induction of IRF3 and IRF7 by different concentrations of fumaric acid detected after GCRV infection of CIK cells. (* *p* < 0.05, ** *p* < 0.01, *** *p* < 0.001).

Analysis of both the proteomic and metabolomic data of this study showed that fumaric acid involved in the butanoate metabolism is downregulated during GCRV infection (*p* = 7.33 × 10^−5^), with a significantly lower *p* value in comparison to other metabolites, such as maleic acid (*p* = 1.09 × 10^−3^) and pyruvic acid (*p* = 1.41 × 10^−2^). Previous studies have also identified fumaric acid as a signaling metabolite with innate immunomodulatory functions [15,16,17]. Therefore, in the present study we treated CIK cells with different concentrations of fumaric and GCRV to determine whether fumaric acid could inhibit GCRV replication. The effects of different concentrations of fumaric acid on CIK cell viability are shown in Supplementary Figure S3D; however, none of the concentrations used affected cell viability. Therefore, we pretreated CIK cells with 0–0.8 mg/mL fumaric acid prior to GCRV infection. The results showed that the content of GCRV in the high concentration (0.6 mg/mL, 0.8 mg/mL) fumaric acid-treated group was extremely significantly decreased compared with that of the control group 24 h after GCRV infection (*p* < 0.0001), whereas there was no significant change in the content of GCRV in the low concentration (0.1 mg/mL, 0.2 mg/mL, 0.4 mg/mL) fumaric acid-treated groups (Figure 5A), suggesting that fumaric acid concentration has an important effect on its anti-GCRV function.

We subsequently explored the feasibility of using fumaric acid as a feed additive. Fumaric acid-supplemented and normal feed were provided to starved juvenile grass carp for 15 consecutive days. Following GCRV infection, the fumaric acid-supplemented group showed a reduced mortality rate compared to the normal feed group, showing an earlier end of pathogenesis, while the average survival rate increased by 19.60% (Figure 5B). H&E staining of the tissue showed that, before GCRV infection, there were no obvious differences between the tissues of the fumaric acid-supplemented and control groups. After GCRV infection, the cell gap in liver and spleen cells increased, and liver cells underwent pyknosis. In addition, the number of monocytes was increased and the renal tubules were constricted in the kidney. However, the lesions in the fumaric acid-supplemented group were less severe than those in the control group. (Figure 5C).

To further investigate the anti-GCRV mechanisms of fumaric acid, we measured the relative expression levels of IFN and IRF in cells. During GCRV infection, high concentrations of fumaric acid induced the simultaneous upregulation of IFN1, IFN3, IRF3, and IRF7, with a higher upregulation of IFN1 and IRF7. However, low concentrations of fumaric acid inhibited IFN and IRF expression (Figure 5A,D). These results demonstrate that the concentration of fumaric acid is a key factor influencing the interferon system. In summary, high concentrations of fumaric acid may inhibit GCRV infection by inducing interferon expression.

## 3. Discussion

Hemorrhagic disease caused by GCRV infection is an urgent problem in grass carp aquaculture; identifying the key factors and pathways affected by GCRV infection could provide ideas for the control of grass carp hemorrhagic disease. Currently, proteomic studies on GCRV infection are rare, although some have been reported in CIK cells [5], rare minnow [7], and grass carp [6], while metabolomic studies have only been reported in grass carp and rare minnow [8,9]. These studies detected approximately 792-2832 proteins and 516-1941 metabolites with quantitative information, whereas our study identified significantly more proteins (10,808) and metabolites (4040). This study should therefore provide a more comprehensive basis for data analysis to understand the changes in proteins and metabolic pathways induced by GCRV infection in grass carp at a holistic level.

The DEP biological processes, DEM classes, and KEGG pathways in this study all showed co-enrichment in the carbohydrate and lipid metabolism, indicating that GCRV predominantly affects these two types of metabolic pathways in the host. However, the specific metabolic pathways that were affected varied across the tissues. For example, arachidonic acid was downregulated in the gills (FC = 0.27, *p* < 0.05); lysosomal hydrolases were upregulated in the liver; while intestinal glycine (FC = 0.34, *p* < 0.05), serine (FC = 0.42, *p* < 0.05), and threonine (FC = 0.41, *p* < 0.05) were all downregulated. Arachidonic acid is a key inflammatory intermediate [18], and activation of the lysosomal pathway implies the onset of a range of biomolecular degradation reactions [19], while glycine, serine, and threonine metabolism affects complement-mediated killing effects [20]. It was thus inferred that the inflammation-associated immune system in the gills was disrupted, a strong lysosomal autophagy reaction occurred in the liver, and the complement function in the intestine was disrupted following GCRV infection. In addition, all three metabolic pathways enriched in the kidney were mitochondria-related; among these, the DEPs and DEMs of the butanoate metabolism were concentrated in the TCA cycle, while nicotinamide was downregulated (FC = 0.70, *p* < 0.01). Nicotinamide, a precursor of nicotinamide adenine dinucleotide (NAD), can influence many important metabolic pathways, such as glycolysis, fatty acid β-oxidation, and oxidative phosphorylation [21]. Conversely, the differential metabolites in muscle were found to be concentrated in amino acids (histidine metabolism), sugars (galactose metabolism, glycolysis), lipids (glycerophospholipid metabolism, tyrosine metabolism), and nucleotide metabolism. It was also inferred that the mitochondrial function of the kidney was severely impaired, while the homeostasis of carbohydrate and lipid metabolism was disrupted in the kidney and muscle following GCRV infection.

The glycolysis and pentose phosphate pathways, which belong to the carbohydrate metabolic pathway, showed highly significant enrichment at both the protein and metabolic levels (*p* < 0.001). Prior research has further shown that infection with some viruses, including white spot syndrome virus (WSSV) [22], human immunodeficiency virus type 1 (HIV-1) [23], severe acute respiratory syndrome coronavirus 2 (SARS-CoV-2) [24], and infectious spleen and kidney necrosis virus (ISKNV) [25], can promote glucose entry into the pentose phosphate pathway. With the enhancement of the pentose phosphate pathway, viruses can further utilize the large number of nucleotides provided by the non-oxidizing branch of the pathway for replication [26]. Many viruses, such as influenza A virus (H1N1) [27] and coxsackievirus B3 [28], can also induce glycolysis of the host to assist in viral replication, while glycolysis can also affect the pentose phosphate pathway through glucose-6-phosphate [26]. In this study, we found that the inhibition of both glycolysis and pentose phosphate metabolic pathways in CIK cells significantly inhibited GCRV replication, indicating that glycolysis and the pentose phosphate pathways in grass carp are important for GCRV proliferation.

Lipid metabolism is essential for viral replication, and lipids have been shown to play an important role in the fusion of viral and host cell membranes, viral replication, endocytosis, and release of viruses, as well as a role in the maturation of viral proteins and synthesis of viral envelopes [29,30]. Fatty acid synthesis is an important step in lipid metabolism, and is required for the replication of viruses such as SARS-CoV-2 and Singapore grouper iridovirus (SgIV) [31,32]. Fatty acid synthesis must be accomplished using citric acid, an intermediate product of the TCA cycle; thus, viral replication is highly dependent on the TCA cycle [33,34]. In the present study, we added DCA to promote TCA cycling prior to viral infection, which has been shown to inhibit GCRV replication; however, DCA was found to significantly promote GCRV replication when DCA was added after viral infection. While it has been shown that promoting TCA cycling inhibits viral replication [25,35], it has also been found that promoting TCA cycling in the absence of viral infection promotes the synthesis of intermediates, such as succinate and fumarate [36,37]. We inferred that these intermediates could have enhanced host innate immunity in the present study, which led to a decrease in viral replication. In our study, by the time DCA was added following viral infection, the virus had already pre-hijacked the host TCA cycle to synthesize its own raw materials; as such, facilitating the TCA cycle at this time would promote fatty acid synthesis and lead to an increase in the level of viral replication.

In the present study, we found that the number of DEPs and DEMs in the kidney before and after infection was significantly higher than in other tissues, suggesting that the kidney of grass carp changes more and responds to a greater extent at the protein and metabolite levels after infection with GCRV. Because the host carbohydrate metabolism was more affected by GCRV infection, we conducted a further study on butanoate metabolism, the only carbohydrate metabolic pathway in which DEPs and DEMs in the kidney were co-enriched. Overall, we found that fumaric acid, involved in butanoate metabolism, was significantly downregulated (*p* < 0.0001). It has further been shown that fumaric acid can activate natural immunity and promote the production of cytokines, such as the pro-inflammatory cytokines TNF and type I interferon (IFN1) [16,37], Dimethyl fumarate (DMF), a derivative of fumaric acid, can also inhibit the replication of HIV and SARS-CoV-2 [38,39]. Furthermore, fumaric acid in the liver of tilapia was downregulated following infection with *Edwardsiella spp.* [40]. To verify whether fumaric acid could influence the immune response of grass carp, we added fumaric acid to the cell culture medium prior to GCRV infection. Our results showed that fumaric acid (>0.6 mg/mL) induced a significant up-regulation of IFN1 and IRF7, as well as significantly inhibiting the replication of GCRV. Fumaric acid realizes its function depending on its accumulation in cells [41]. We also found that only high concentrations of fumaric acid (>0.6 mg/mL) exerted antiviral effects, whereas low concentrations of fumaric acid (<0.6 mg/mL) decreased IFN and IRF expression. IRF is an important transcription factor involved in the antiviral immune response to IFN, which can initiate the transcriptional expression of IFN [42]. Furthermore, transcriptome studies of resistant and susceptible grass carps showed that the IFNs were closely related to anti-GCRV infection [5]. These results imply that high concentrations of fumaric acid in grass carp may exert antiviral functions by upregulating the expression of IRF7 to enhance IFN1 transcription. Fumaric acid is a commonly used feed additive for swine and poultry, and its addition to juvenile African catfish and Nile tilapia feed has been observed to induce many benefits, such as promoting growth, improving feed utilization efficiency, and improving the survival of mild Aeromonas infection in African catfish [43,44]. However, there have been no prior reports on the antiviral function of fumaric acid as an additive; therefore, further studies in juvenile grass carp showed that the addition of fumaric acid to feed increased the survival rate, slowed down the rate of death, and had a protective effect on tissues, making it a potential anti-GCRV feed additive.

Currently, vaccines and antiviral agents are the main therapeutic measures for GCHD [45,46]. Here, we found that fumaric acid can be used as an anti-GCRV feed additive, which is economically friendly, chemically stable, and easy to handle compared to vaccines. However, because of the differences in gene and protein sequences between GCRV strains and the high lethality of GCRV [45,47], no therapeutic intervention has been successful in eradicating GCHD in grass carp culture. In the future, we can optimize preparation methods of fumaric acid feed and explore the role of fumaric acid content on GCRV resistance in juvenile grass carp. Furthermore, the signaling pathway regulating fumaric acid can be gene-edited in the hope of producing GCRV-resistant varieties.

## 4. Materials and Methods

### 4.1. Grass Carp, Cells, Viruses and Ethics

The grass carp used in this experiment were all full-sibling six-month-old grass carp (15 ± 3 cm, 40 ± 10 g), provided by the Guanqiao Experimental Base at the Institute of Hydrobiology, Chinese Academy of Sciences. The fish were pre-cultured in water at 27–28 °C and fed twice a day. After one week, the experimental fish were confirmed to be free from health problems (ate the feed and swam normally, with no deaths or obvious damage to the body surface), after which they were infected with type II GCRV using the method designed in our laboratory [48].

The frozen CIK cells were purchased from China Model Culture Conservation Center (CCTCC) and delivered on dry ice. Then, the cells were recovered and cultured in a constant temperature incubator at 28 °C with a 5% CO_2_ concentration in M199 medium supplemented with 10% fetal bovine serum, 100 U/mL penicillin, and 100 g/mL streptomycin. For viral infection, CIK cells (3-40 generation) were first inoculated in 6-well plates; when the growing area reached 80% on the second day, fresh complete medium containing GCRV (5 × 10^5^ TCID50/mL) was replaced for infection. The cells were collected, and RNA was extracted after 24 h. The virulent GCRV strains were prepared and preserved in our laboratory [49].

All feeding, sampling, and viral infections of the experimental fish were performed in accordance with the relevant policies and regulations of the Guidelines for the Care and Use of Laboratory Animals (Ministry of Science and Technology of China, 2006), while all experimental procedures were approved by the Ethics Committee of the Institute of Hydrobiology, Chinese Academy of Sciences (E311031001).

### 4.2. Tissue Sample Collection

Tissue samples from the experimental group were collected when grass carp were infected with type II GCRV and showed obvious symptoms, such as straying and swimming alone, or swimming imbalance. Five tissue samples were collected from each fish, including gill (G), liver (L), intestine (I), kidney (K), and muscle (M), and 100 mg of each tissue was sampled. Healthy grass carp with the same genetic background were used as a control group for simultaneous collection of tissue samples. To eliminate the effects of individual differences as much as possible, the same tissue samples from three fish in the experimental and control groups were mixed into one sample (~300 mg) for sequencing, while three parallel samples were prepared for each sample to obtain a total of 30 samples (5 × 3 × 2). All samples were frozen in liquid nitrogen immediately after collection. Some samples were retained for RNA extraction, with subsequent gene expression and tissue viral load detection. The primer sequences for fluorescence quantitative PCR (qPCR) of the type II GCRV S6 segments are shown in Supplementary Table S11.

### 4.3. DIA Proteomic and Untargeted Metabolomic

Sample extraction, DIA proteomics, and untargeted metabolomics were performed by Shenzhen BGI Genomics Co., Ltd., Shenzhen, China.

DIA proteomic liquid phase separation was performed on a Shimadzu LC-20AD liquid phase system (Shimadzu, Kyoto, Japan), while DDA library construction and DIA sample analysis was performed on a high-performance liquid chromatograph (UltiMate 3000 UHPLC, Thermo Fisher Scientific, San Jose, CA, USA) and a mass spectrometer (Orbitrap Exploris 480, Thermo Fisher Scientific, San Jose, CA, USA). Sample liquid-phase separation, DDA library construction, and DIA analysis were performed as previously described [50]. The DDA data library was used as a spectral library for DIA analysis using MaxQuant software (version 1.5.3.30). Database searches were performed using self-constructed grass carp protein database. Spectronaut was used to analyze the DIA data, and iRT peptides were used to correct retention times. A false-positive control was then made with a false discovery rate (FDR) of 1%, based on the target-decoy model applied by SWATH-MS. Finally, the MSstats was used to statistically assess the differences in expression between the different tissue proteomes. Error correction was further applied to each sample, after which normalization was performed, and significant difference protein screening was performed based on fold change (FC) ≥ 2 and *p* < 0.05. The proteins were annotated according to the non-redundant and SWISS-PROT databases.

Ultra-performance liquid chromatography-mass spectrometry (UPLC-MS) analysis of untargeted proteomics was performed using a Waters 2777C UPLC (Waters, Milford, MA, USA), in tandem with a Q Exactive HF high-resolution mass spectrometer (Thermo Fisher Scientific, USA) in ESI mode for metabolite separation and detection. Mass spectrometry data were imported into the Compound Discoverer 3.3 (Thermo Fisher Scientific, San Jose, CA, USA) and analyzed using the BMDB (BGI Metabolome Database), mzCloud, and ChemSpider online databases to yield a data matrix containing the metabolite peak areas and identification results. The data matrix containing information on the metabolite peak areas and identification results was thus obtained. Subsequently, the results exported from Compound Discoverer were imported into metaX for data preprocessing, and the data were normalized using Probabilistic Quotient Normalization (PQN) to obtain the relative peak areas. Quality control-based robust LOESS signal correction (QC-RLSC) was further applied to correct for batch effects, and compounds with a relative peak area Coefficient of Variation (CV) greater than 30% were removed from all QC samples. The resulting data matrix was used for subsequent analysis. Metabolomic data were then analyzed using univariate analysis to calculate the Fold change, while Student’s t test was applied to calculate the *p* value, and screened for significantly different metabolites based on FC ≥1.2 or ≤0.83 and *p* < 0.05.

### 4.4. Treatment of CIK Cells with Inhibitors

The inhibitors used were 2-DG (Selleck, Houston, TX, USA), DHEA (MedChemExpress, Monmouth Junction, NJ, USA), and DCA (Selleck, Houston, TX, USA), which act as an inhibitor of glycolysis [51], an inhibitor of the pentose phosphate pathway (PPP) [52], and a promoter of the entry of pyruvate into the tricarboxylic acid (TCA) cycle [53], respectively. The 2-DG was dissolved in water to yield a 10 mg/mL master mix, and DHEA and DCA were dissolved in DMSO to obtain 10 mM master mixes, respectively.

The effect of inhibitors on cell viability was determined prior to performing inhibitor treatment. To achieve this, CIK cells were cultured in 96-well plates; when 80% confluency was reached on the second day, the medium was replaced with complete medium containing different final concentrations of 2-DG (0, 0.1, 0.2, 0.4, 0.6, 0.8 mg/mL), DHEA (0, 1, 2, 5, 10, 20 µM), and DCA (0, 1, 2, 5, 10, 20 µM), after which cells were cultured for a further 24 h. A final concentration of 0 was the corresponding dissolution reagent of the drug, which served as the control. Cell viability was subsequently determined using the CCK8 assay kit (Beyotime, Zhenzhou, China), according to the manufacturer’s instructions.

Cells in 6-well plates were pretreated with complete medium containing 2-DG, DHEA, and DCA for 4 h prior to viral infection, with dimethyl sulfoxide (DMSO) used as a control. Additionally, cells were treated with DCA at 6 h after viral infection. The cell viral infection experiments are described in Section 2.1. RNA extraction, reverse transcription and fluorescence quantitative PCR were performed to detect the replication level of GCRV S6 segment after 24 h of infection, and the primer sequences for fluorescence quantification PCR of type I GCRV S6 segment are shown in Supplementary Table S11.

### 4.5. Fumaric Acid Treatment of Cells and Grass Carp Juveniles

Fumaric acid (Shanghai Yuanye Bio-Technology Co., Ltd., Shanghai, China) was dissolved in DMSO to form a 10 mg/mL master mix. The effects of fumaric acid on cell viability were determined. In brief CIK cells were cultured in 96-well plates; when the confluency reached 80% on the second day, the medium was replaced with complete medium containing different final concentrations of fumaric acid (0, 0.1, 0.2, 0.4, 0.6, and 0.8 mg/mL) and cultured for 24 h. DMSO was used as a 0 mg/mL control. Subsequently, cell viability was determined using the CCK8 kit (Beyotime, Zhenzhou, China), according to the manufacturer’s instruction. Cells in 6-well plates were pretreated with fumaric acid for 4 h prior to viral infection, and DMSO was used as a control. The cell viral infection experiments are described in Section 2.1. RNA extraction, reverse transcription, and fluorescence quantitative PCR were performed to detect the replication level of the type I GCRV S6 segment and the expression of interferon (IFN) and interferon-related (IRF) genes after 24 h of infection. The sequences of the primers used for the fluorescence quantitative PCR of IFN and IRF are shown in Supplementary Table S11.

A 1% feed mass of fumaric acid was fully dissolved in ethanol, mixed with puffed feed containing 30% protein, and dried naturally. The control group was fed normal feed and the experimental group was fed fumaric acid feed. Three parallel groups were established for each group, containing approximately 50 fish per group. Fish were fed twice a day with 5% of fish’s body weight, once in the morning and once in the evening, and infected with the virus after 15 consecutive days of feeding. The viral infection method is described in Section 2.1. The experiment was considered complete if no dead individuals were found after one week. Samples of the liver, spleen, and kidney were randomly selected from healthy and virus-infected fish, sent to Icongene Biotechnology Co., Wuhan, China and fixed in 4% paraformaldehyde for tissue sectioning and hematoxylin-eosin (H&E) staining.

### 4.6. RNA Extraction, Reverse Transcription PCR (RT-PCR) and Real-Time Fluorescence Quantitative PCR (qPCR)

All samples were extracted using TRIzol reagent (Fisher Scientific, San Jose, CA, USA). Tissue samples were ground by tissue grinder (Shanghai JingXin, Shanghai, China) in 1 mL TRIzol with grinding beads and cell samples were lysed directly using 1 mL TRIzol. Then 200 µL trichloromethane was added to the TRIzol, mixed thoroughly and left to stand for 2 min, centrifuged at 12,000 rpm at 4 °C for 10 min. A measure of 500 µL supernatant was taken to another Rnase-free centrifuge tube, 500 µL isopropanol was added to the supernatant, mixed well and then precipitated at −20 °C. After 30 min, the mixture was taken out and centrifuged at 12,000 rpm at 4 °C for 15 min, the supernatant was discarded and the sediment washed with 800 µL 75% ethanol. Then, it was centrifuged at 12,000 rpm for 5 min at 4 °C and the supernatant discarded. It was dried for 5–10 min and 50 µL Rnase-free water added. Finally, quality control was performed using 1% agarose electrophoresis and the concentration determinatied.

The reverse transcription was performed using the Vazyme HiScript II 1st Strand cDNA Synthesis Kit (Vazyme, Nanjing, China) with 1 µg RNA. RT-PCR system was configured using 2 × Taq Master Mix (Dye Plus) (Vazyme, Nanjing, China). The RT-PCR primer sequences are shown in Supplementary Table S12. qPCR system was configured using Vazyme HiScript II Q RT SuperMix for qPCR +gDNA wiper (Vazyme, Nanjing, China) with diluted cDNA. Primers of genes identified on proteomic analysis were designed using the Primer 3 online website, and the qPCR primer sequences are shown in Supplementary Table S11. All of the above experimental steps were performed according to the manufacture. The qPCR was performed using a real-time fluorescence quantitative PCR instrument (CFX96, Bio-Rad, Hercules, CA, USA), with three replicates for each sample. The β-actin gene was used as an internal reference for normalization of gene expression. The relative expressions of GCRV and genes in grass carp tissues and cells were calculated using the 2-ΔΔCt method [54]. GCRV copies in the cell supernatants were calculated using an absolute quantitative method [55]. In brief, the GCRV S6 segment was cloned into the PMD18-T vector (Takara, Osaka, Japan) and subjected to gradient dilution. qPCR was further performed with gradient dilution samples to obtain a standard curve of Ct values versus copies, and the corresponding copies were calculated according to the Ct value of the sample to be tested.

### 4.7. Bioinformatics Analysis and Statistical Analysis of Data

Proteomic and metabolomic data were first subjected to principal component analysis (PCA) to compare intragroup and intergroup differences, after which the numbers of DEPs and DEMs were counted in each comparative group, followed by Gene Ontology (GO) and Kyoto Encyclopedia of Genes and Genomes (KEGG) pathway analyses.

qPCR results are expressed as the mean and standard deviation (SD) of at least three experiments. Statistical differences between the groups were determined using an independent sample t-test, performed using SPSS software (version 21.0; IBM, New York, NY, USA). *p* < 0.05 was considered as statistically significant, while *p* < 0.01 was considered as highly significant.

## 5. Conclusions

In this study, we used DIA proteomics and untargeted metabolomics to identify DEPs and DEMs in five tissue types from grass carp before and after GCRV infection, finding that the kidney was the most affected tissue. Combined proteomic and metabolomic analyses revealed that GCRV primarily affected the carbohydrate and lipid metabolism in the host. Considering the shared metabolic pathways in each tissue, combined with the results of the glycolysis and pentose phosphate pathway inhibitor treatment experiments, we inferred that the host glycolysis and pentose phosphate pathways play important roles in GCRV replication. Finally, we analyzed butanoate metabolism in the kidney and found that high concentrations of fumaric acid inhibited GCRV replication by inducing IFN1 expression at the cellular level. In addition, fumaric acid protected tissues and increased the survival rate of grass carp juveniles during GCRV infection, making it a promising anti-GCRV feed additive.

## Data Availability

The proteomic raw data have been submitted to the Integrated Proteome Resources (iProX) database (https://www.iprox.cn/ accession number: IPX0009842001). The metabolomic raw data have been submitted to Metabolights database (https://www.ebi.ac.uk/metabolights/ accession number: MTBLS11400, accessed on 26 September 2024).

## Supplementary Materials

The following supporting information can be downloaded at: https://www.mdpi.com/article/10.3390/ijms252111852/s1.
