# Supplementary material for: Analysis of GCRV Pathogenesis and Therapeutic Measures Through Proteomic and Metabolomic Investigations in GCRV-Infected Tissues of Grass Carp (Ctenopharyngodon idella)
Source: Int J Mol Sci. 2024 Nov 4;25(21):11852. doi: 10.3390/ijms252111852 (PMC11546743; doi:10.3390/ijms252111852)
Supplement: Supplementary file 1 [file ijms-25-11852-s001.zip › Supplementary Figures.pdf]

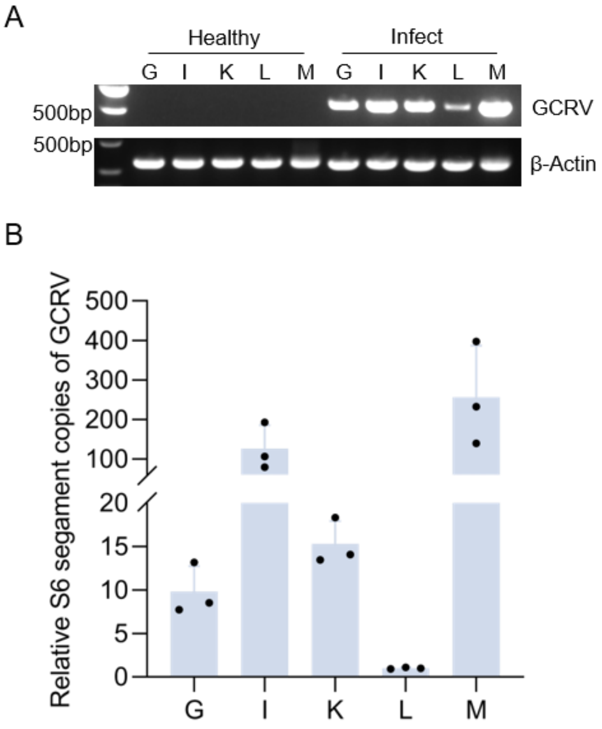

Figure S1. Relative viral load in five tissues. (A) Detection of viral infections in healthy and infected fish using reverse transcription PCR (RT-PCR), beta-actin as a reference gene. (B) Relative viral load in five tissues after GCRV infection.

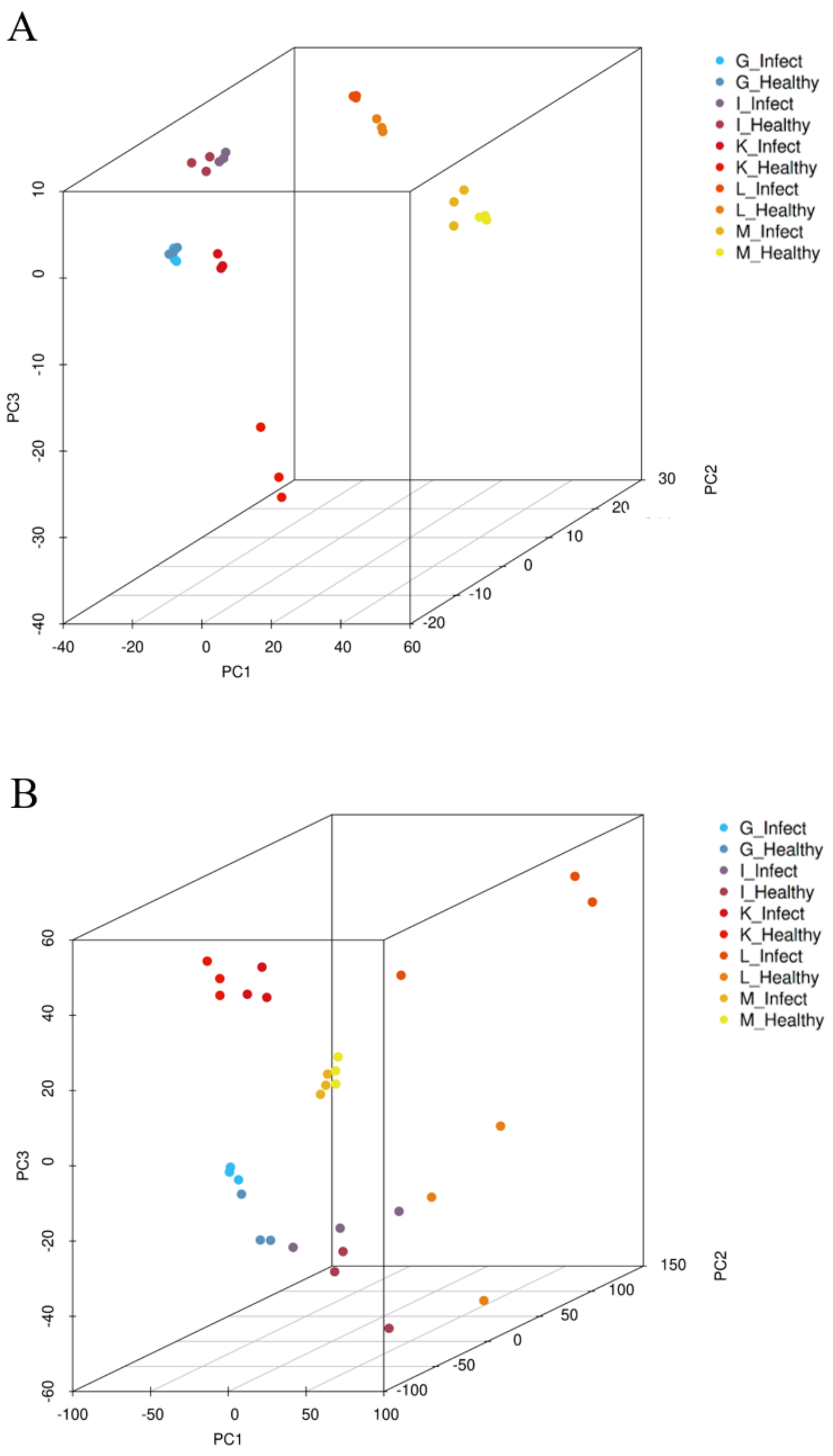

Figure S2. Principal component analysis. (A) Principal component analysis of proteomic data. (B) Principal component analysis of metabolomic data.

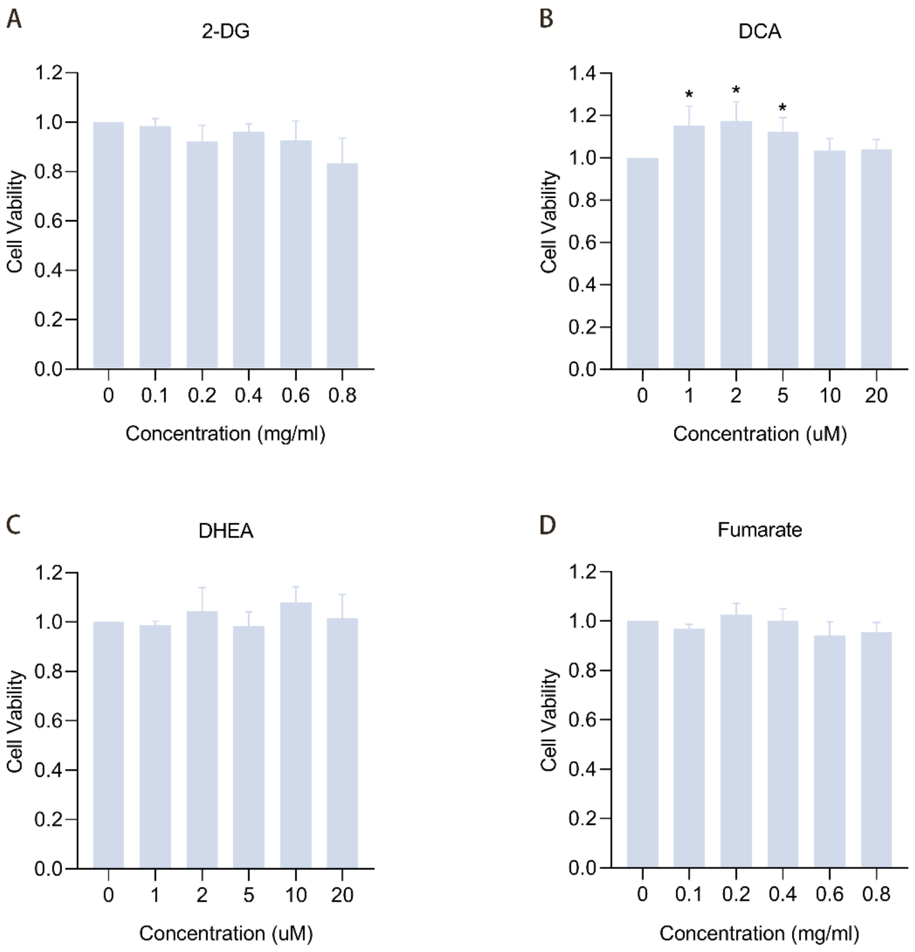

Figure S3. CIK Cell Activity Assay. (A) CIK cell viability treated with different concentrations of 2-DG. (B) CIK cell viability treated with different concentrations of DCA. (C) CIK cell viability treated with different concentrations of DHEA. (B) CIK cell viability treated with different concentrations of Fumarate.
