# Supplementary material for: Analysis of GCRV Pathogenesis and Therapeutic Measures Through Proteomic and Metabolomic Investigations in GCRV-Infected Tissues of Grass Carp (Ctenopharyngodon idella)
Source: Int J Mol Sci. 2024 Nov 4;25(21):11852. doi: 10.3390/ijms252111852 (PMC11546743; doi:10.3390/ijms252111852)
Supplement: Supplementary file 1 [file ijms-25-11852-s001.zip › Supplementary Table S11.pdf]

Supplementary Table S11 Primer sequences for qPCR analysis

Target genes	Sequence (5'-3')	Product size (bp)
GCRV Ⅰ S6	CCTGTGCCTCACCTACCCAAA<br>GAACCAACGAGACTCCCAACTTC	171
GCRV Ⅱ S6	CGGGACAACATCCAAGACGA<br>AGCATCAGCAATGCAGGAGT	186
AAS89353.1	TTCGGCTGATGTCTCTGGTT<br>CCCTCCAAAAGTTGAGCCAC	161
KTF81920.1	ACCTGCCTCATCAAGTCACT<br>TCCTGTCAAACCATCTGCCA	111
XP_016371210.1	GTTTAACGCGTACCTCCTGC<br>GGGTCTGTTGAAGGTCCTGA	172
XP_005158243.1	GCATCTCATCCCAAACCGTT<br>TCTTGCTCCCACGGAAATCT	102
NP_997783.1	AGTGTGTGACCTCTGGATGG<br>AGTCAGTGTTGGTCAGCAGA	99
XP_016324649.1	ACTTTGTCCGCCAGTACAGT<br>GCATTGCCGTACATGACCAT	130
β-actin	GATATGGAGAAGATCTGGCA<br>CTGTTGGCTTTGGGGTTCAG	107
IFN1	AAGCAACGAGTCTTTGAGCCT<br>GCGTCCTGGAAATGACACCT	79
IFN3	TACATTTATAGAGACTGCGGGTGG<br>TGGAGTGTCTGGTAAACAGCCTT	144
IRF3	ACTTCAGCAGTTTAGCATTCCC<br>GCAGCATCGTTCTTGTTGTCA	207
IRF7	CGCCTGTGTTCGTCACTCGT<br>GGTGGTTGGAAAGCGTATTGG	84
