# Supplementary material for: Analysis of GCRV Pathogenesis and Therapeutic Measures Through Proteomic and Metabolomic Investigations in GCRV-Infected Tissues of Grass Carp (Ctenopharyngodon idella)
Source: Int J Mol Sci. 2024 Nov 4;25(21):11852. doi: 10.3390/ijms252111852 (PMC11546743; doi:10.3390/ijms252111852)
Supplement: Supplementary file 1 [file ijms-25-11852-s001.zip › Supplementary Table S12.pdf]

Supplementary Table S12 Primer sequences for RT-PCR analysis

Target genes	Sequence (5'-3')	Product size (bp)
GCRV Ⅱ S6	GGCGGCTGCTATGATACCAGTAGAC<br>GTCGCTTCAGATCAATCACCAAATC	539
β-actin	TCGGTATGGGACAGAAGGAC<br>GACCAGAGGCATACAGGGAC	306
